# Effect of Hydrolyzed Bird’s Nest on β-Cell Function and Insulin Signaling in Type 2 Diabetic Mice

**DOI:** 10.3389/fphar.2021.632169

**Published:** 2021-04-13

**Authors:** Ker Woon Choy, Zuhaida Md Zain, Dharmani Devi Murugan, Nelli Giribabu, Nor Hisam Zamakshshari, Yang Mooi Lim, Mohd Rais Mustafa

**Affiliations:** ^1^ Department of Anatomy, Faculty of Medicine, Universiti Teknologi MARA, Selangor, Malaysia; ^2^ Department of Pharmacology, Faculty of Medicine, University of Malaya, Kuala Lumpur, Malaysia; ^3^ Department of Physiology, Faculty of Medicine, University of Malaya, Kuala Lumpur, Malaysia; ^4^ Centre for Natural Product Research and Drug Discovery (CENAR), Wellness Research Cluster, University of Malaya, Kuala Lumpur, Malaysia; ^5^ Department of Pre-Clinical Sciences, Centre for Cancer Research, Faculty of Medicine and Health Sciences, University Tunku Abdul Rahman, Selangor, Malaysia

**Keywords:** hydrolyzed bird nest, type 2 diabetes mellitus, insulin signaling, oxidative stress, inflammation

## Abstract

Type 2 diabetes mellitus is characterized by both resistance to the action of insulin and defects in insulin secretion. Bird’s nest, which is derived from the saliva of swiftlets are well known to possess multiple health benefits dating back to Imperial China. However, it’s effect on diabetes mellitus and influence on the actions of insulin action remains to be investigated. In the present study, the effect of standardized aqueous extract of hydrolyzed edible bird nest (HBN) on metabolic characteristics and insulin signaling pathway in pancreas, liver and skeletal muscle of *db/db*, a type 2 diabetic mice model was investigated. Male *db/db* diabetic and its euglycemic control, C57BL/6J mice were administered HBN (75 and 150 mg/kg) or glibenclamide (1 mg/kg) orally for 28 days. Metabolic parameters were evaluated by measuring fasting blood glucose, serum insulin and oral glucose tolerance test (OGTT). Insulin signaling and activation of inflammatory pathways in liver, adipose, pancreas and muscle tissue were evaluated by Western blotting and immunohistochemistry. Pro-inflammatory cytokines were measured in the serum at the end of the treatment. The results showed that *db/db* mice treated with HBN significantly reversed the elevated fasting blood glucose, serum insulin, serum pro-inflammatory cytokines levels and the impaired OGTT without affecting the body weight of the mice in all groups. Furthermore, HBN treatment significantly ameliorated pathological changes and increased the protein expression of insulin, and glucose transporters in the pancreatic islets (GLUT-2), liver and skeletal muscle (GLUT-4). Likewise, the Western blots analysis denotes improved insulin signaling and antioxidant enzyme, decreased reactive oxygen species producing enzymes and inflammatory molecules in the liver and adipose tissues of HBN treated diabetic mice. These results suggest that HBN improves β-cell function and insulin signaling by attenuation of oxidative stress mediated chronic inflammation in the type 2 diabetic mice.

## Introduction

Diabetes mellitus is a chronic metabolic disorder of endocrine system which can be categorized into two types: type 1 diabetes (insulin dependent diabetes mellitus) and type 2 diabetes (T2DM, non-insulin dependent diabetes mellitus). T2DM account for 90% of the clinical cases and is the most prevalent form of diabetes (Domingueti et al., 2016). In 2019, approximately 463 million adults (20–79 years) are living with diabetes and this figure is expected to rise to 700 million by 2045 (International Diabetes Federation, 2019). The characteristics of the T2DM are peripheral insulin resistance, insulin insensitivity, decreased β-cell function, and impaired regulation of hepatic glucose production which ultimately leads to pancreatic β-cell failure (Halim and Halim, 2019). The development of T2DM is positively linked with chronic low levels of inflammation (Lontchi-Yimagou et al., 2013).

Insulin, the main hormone that is capable of reducing the blood glucose level, interacts with its receptor to activate an intrinsic tyrosine protein kinase, which autophosphorylates the receptor as well as Insulin Receptor Substrates (IRS). The IRS then activates a cascade of serine-protein kinases which includes Akt (protein kinase B). Akt is a major branch point with numerous downstream substrates leading to a variety of physiological functions including the regulation of energy homeostasis (Beale, 2013). Besides the liver and pancreas, adipose tissue also regulates metabolism by regulating insulin sensitivity in target tissues by several ways 1) storing fat as triglyceride and releasing it as fatty acids and glycerol as needed; and 2) releasing a variety of hormones, collectively known as adipokines (Beale, 2013).

Currently, drugs such as metformin, glibenclamide and thiazolidinediones are mainly used for clinical management of diabetes (Ahren et al., 2017). However, prolonged use of these drugs are associated with long-term side effects such as liver and kidney damage (Prescrire International, 2014). Therefore, there is an on-going need to develop anti-diabetic agents with better efficacy and less adverse effects.

Recently, natural products have gained attention in clinical medicine as a potential treatment in management of type 2 diabetes mellitus due to their improved efficacy and less side effects (Li et al., 2017). Edible bird’s nest is an Asian traditional food supplement known for its nutritional value and is believed to enhance energy levels, prevent aging and improve overall health (Vimala et al., 2012). It’s also been proven in several studies as good source of antioxidants, is anti-inflammatory, and desirable bone-strengthening effects (Matsukawa et al., 2011; Vimala et al., 2012; Yew et al., 2014; Yida et al., 2014). Furthermore, Yida et al., showed that edible bird's nest prevents high-fat diet induced insulin resistance in rats (Yida et al., 2015). Recently, we have demonstrated the vascular protective effect of hydrolyzed bird’s nest in hyperglycaemic condition (Murugan et al., 2020). However, not much is known on the effect of edible bird nest in type 2 diabetes. Therefore, in the present study, the anti-diabetic effects and mechanism of hydrolyzed bird’s nest (HBN) is investigated through measurement of general and metabolic parameters, protein expression and morphology changes in liver, adipose, pancreas and kidney to provide further a basis for the use of HBN as a potential nutraceutical supplement in type 2 diabetes mellitus.

## Materials and Methods

### Preparation of HBN and Chemical Profiling of HBN

The HBN was prepared and kindly provided by Professor Lim Yang Mooi from University Tunku Abdul Rahman, Malaysia. A voucher specimen was deposited in the Nature Inspired UM Natural Products Library, University Malaya (voucher number UMCNA1801). Briefly, the raw edible birds nest from Aerodramus fuciphagus (white nest) from swiftlet houses was cleaned and made to powder. The raw cleaned EBN powder was then suspended in distilled deionized water with the ratio of 0.2% (w/v) for 24 h at 4°C. The mixture was then heated at 80°C in the distilled water for an hour. The extracts were allowed to cool and further centrifuged at 2,700 *g* to collect the supernatant for subsequent lyophilization. The dried extract was stored at −20°C until further analysis. Chemical profiling of HBN was carried out using LCMS-QTOF. Solvents system in LCMS consist of 0.1% formic acid in water (A) and acetonitrile (solvent B) were used with the following gradient: starting with 100% B and reduction to 50% B at 18 min, and finally 5% B from 18 to 30 min. The system controller was stopped at 20 min. The solvent’s flow rate was 0.8 ml/min. Samples (10 μl) were injected in to a C18 reversed-phase column (150 × 4.6 mm i.d, 3.0 µm particle size). Mass spectrometric detection was performed with a quadrupole-TOF-MS operated in the positive mode. Information dependent acquisition was conducted using a TOF-MS survey scan 100–1,100 Da (100 ms) and up to 10 dependent TOF MS scans 100–1,100 Da (100 ms) with Collision Energy (CE) of 45 V with Collision Energy Spread (CES) of ± 30 V. The identification of the peaks was conducted with Metlin database.

### Animal Preparation

Healthy *db/db* and C57BL/6J male mice (8 weeks old) were purchased from Jackson Laboratory and Monash University (Sunway Campus, Malaysia), respectively. The mice were allowed 2 weeks for acclimatization to the housing facility. All the experimental procedures were approved by the University of Malaya Animal Care and Ethics Committee (Ethics Reference No: 2015–180709) and accredited by Association for Assessment and Accreditation of Laboratory Animal Care International (AAALAC). Animal study was carried out in strict accordance with the established institutional guidelines and the NIH guidelines on the use of experimental animals. The animals were housed in individual ventilated cages with temperature (24 ± 1°C), lighting condition (12 h light/dark cycles), room pressure (10 Pa) and humidity (50–70%) with free access to standard chow (Specialty Feeds Pty Ltd., Glen Forrest, Australia) and tap water ad libitum.

### Experimental Design and Study Protocol

Animals were divided into five groups with six mice in each group as follows:

Group 1: C57BL/6J mice-nondiabetic control mice (received distilled water) *via* oral gavage for 28 days
Group 2: *db/db* mice-control type 2 diabetic mice (received distilled water) *via* oral gavage for 28 days
Group 3: *db/db* mice receiving 75 mg/kg HBN *via* oral gavage for 28 days
Group 4: *db/db* mice receiving 150 mg/kg HBN *via* oral gavage for 28 days
Group 5: *db/db* mice receiving 1 mg/kg glibenclamide (Glib) *via* oral gavage for 28 days (Murugan et al., 2020).

HBN and anti-diabetic agent, glibenclamide (positive control) was dissolved in distilled water. Body weight was measured weekly by using a digital weight scale. Fasting blood glucose (FBG) with blood pricked from tail vein was measured weekly by using a digital glucometer (Accu-Check, Roche Diagnostics). At the end of 28 days, the animals were sacrificed by carbon dioxide (CO_2_) inhalation and blood were collected immediately by cardiac puncture. Liver, pancreas and skeletal muscle were collected for investigating the morphological changes and immunohistochemistry. Some part of the liver and epididymal fat were collected for Western blot.

### Oral Glucose Tolerance Test

Oral Glucose Tolerance Test was performed on overnight fasted animals 2 days before sacrificing the animals at the end of the HBN treatment period. Briefly, glucose load of 3 g/kg of glucose was given orally and blood glucose levels were checked by tail pricking at 0 min (prior to glucose load), 30, 60, 90, and 120 min after loading of glucose (Kunasegaran et al., 2017).

### Determination of Serum Insulin

Blood samples were allowed to clot for 30 min at room temperature, centrifuged at 2,000 rpm for 10 min at 4°C to obtain serum and stored at −80°C immediately until further use. Serum insulin levels were determined using enzyme-linked immunosorbent assay (ELISA) kit (Mercodia Mice Insulin ELISA Kit’s instructions’ (i-DNA Biotechnology) according to the manufacturer’s protocol. In brief, insulin in the sample reacts with peroxidase-conjugated anti-insulin antibodies in the microtiter wells. The samples were washed to remove the unbound enzyme-labeled antibody. The bound conjugate was detected by reaction with 3, 30, 5, 50-tetramethylbenzidine, then stopped with acid which gave a colorimetric endpoint. Optical density was measured by using a micro-plate reader (Tecan, Mannedorf, Switzerland) at wavelength of 450 nm.

### Measurement of Serum Inflammatory IL-6 and TNF-α Levels

The volume/unit of IL6 and TNF-α protein complex were measured in the blood serum by using ELISA kits (Biosource International Inc., Camarillo, CA) following manufacturer’s guidelines. IL-6 and TNF-α were determined from a standard curve and their levels were expressed in pg/ml.

### Histology and Immunohistochemistry

Liver, pancreas and skeletal muscle were harvested and immediately fixed in 10% buffered formalin overnight, then embedded in paraffin and manually cut into 5 μm thick sections by using a microtome (Histo-line laboratories, ARM-3600, Viabrembo, Milan, Italy). Sections were then dewaxed in two changes of xylene, hydrated in two changes of 100% of ethanol, followed by 95 and 80% of ethanol and finally rinsed with H_2_O. Sections were then stained with hematoxylin and eosin (H and E). The stained sections were dehydrated with 80% ethanol followed by 95% ethanol, placed in two changes of 100% of ethanol and cleansed with two changes of xylene. Histopathological changes were viewed by using a phase contrast microscope (Nikon H600L, Nikon DS camera control Unit DS-U2, Version 4.4, Tokyo, Japan), with an attached photograph machine (Nikon H600L).

For immunochemistry, the sections were incubated with 0.01 M citrate buffer, pH 6.0 for 10 min at 100°C and then 3% H_2_O_2_ in phosphate buffered saline (PBS) to neutralize the endogenous peroxidase. Blocking for non-specific binding was done with normal serum (Santa Cruz Biotechnology, Santa Cruz, CA, United States). Following that, sections were incubating with primary polyclonal antibodies for insulin (sc-9168), GLUT-4 (sc-53566) and GLUT-2 (sc-9117) from Santa Cruz Biotechnology, at a dilution of 1:500 in 5% normal serum for 1 h at room temperature. The sections were then rinsed three times with PBS before incubation with biotinylated secondary antibody for 30 min at room temperature, exposed to avidin and biotinylated HRP complex in PBS for another 30 min (ImmunoCruzTM ABC Staining System). 3,30-Diaminobenzidine (DAB) were used to visualize antibody binding site that produced dark-brown precipitate. Sections were counterstained with hematoxylin-eosin (H and E) for nuclear staining.

### Western Blotting

Protein samples obtained from the liver and epididymal fat were lyzed in ice-cold 1X RIPA buffer consists of EGTA 1 mM, EDTA 1 mM, NaF 1 mM, leupeptin 1 μg/ml, aprotinin 5 μg/ml, PMSF 100 μg/ml, sodium orthovanadate 1 mM, and β-glycerolphosphate 2 mg/ml. The lysates were centrifuged, and the supernatant was used for Western blotting. Protein concentrations were quantified using standard Lowry assay protocol by (Bio-Rad Laboratories, Hercules, CA, United States). Twenty micrograms of protein samples were electrophoresed at 100 V through 7.5 or 10% SDS-polyacrylamide gels based on the size of target proteins and transferred to an Immobilon-P polyvinylidene difluoride membrane (Millipore, Billerica, MA, United States). The membranes were incubated with 3% bovine serum albumin (BSA) in 0.05% Tween 20 PBS with gentle shaking to block non-specific binding. The membranes were then incubated with primary antibodies against insulin receptor β (1:1,000, Cell Signaling), p-IRS1 (1:1,000, Cell Signaling), IRS1 (1:1,000, Cell Signaling), p-PI3K (1:1,000, Cell Signaling), p-AKT (1:1,000, Cell Signaling), AKT (1:1,000, Cell Signaling), NFκB (1:1,000, Cell Signaling), NOX4 (1:1,000, Cell Signaling), SOD-1 (1:1,000, Cell Signaling) and GAPDH (1:10,000, Abcam) overnight at 4°C. Following that, the membranes were washed three times with TBS-T and incubated with horseradish peroxidase-conjugated secondary antibodies (DakoCytomation, Carpinteria, CA, United States) at a dilution of 1:10,000 for 1 h. Then, enhanced chemiluminescence detection system (ECL reagents, Millipore Corporation, Billerica, MA) was added onto the membrane exposed on X-ray films and developed by SRX-101 (Konica, Wayne, NJ) for visualization. Quantity One software (Bio-Rad) was used for densitometry analysis. The respective protein expression levels were calculated as ratio of each target band/appropriate internal control and expressed over normal control.

### Statistical Analysis

All results are presented as mean ± standard error of mean (SEM) for the number of rats (*n*) in each group. Data were analyzed for statistical significance using Student’s *t*-test for unpaired observations and, for comparison of more than two groups, one-way ANOVA followed by Bonferroni’s multiple comparison test was performed using the statistical software GraphPad Prism version 4 (GraphPad Software Inc., San Diego, CA, United States); *p* value of less than 0.05 was considered to indicate statistically significant difference.

## Results

### Chemical Profiling of HBN

**FIGURE 1:**
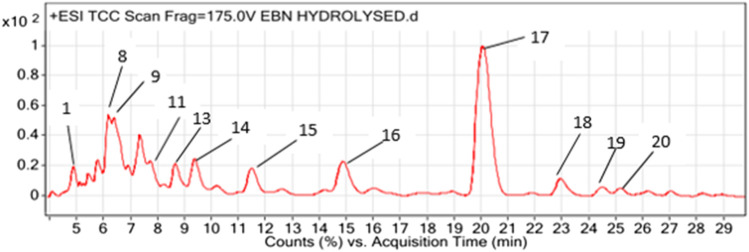
LCMS chromatogram profiling of hydrolyzed bird’s nest (HBN).

Figure 1 shows a LCMS- QTOF chromatogram of HBN. A total of 20 metabolites were identified. Based on the chemical MS library and comparison to the literatures, 17 metabolites (Table 1) were tentatively identified including the bio-marker compounds, sialic acid. Three types of sialic acid were determined in HBN. They are N-Acetylmuramic acid, N-Acetylneuraminic acid and N-Acetyl-2,7-anhydro-alpha-neuraminic acid. The HBN used for this study contained 1.26 µg sialic acid/mg of dried weight (the major active compound in HBN) and it was standardized to contain the same amount of sialic acid for every batch prepared. Other metabolites found were vitamin D and 3-Phenyl-5-ureido-1,2,4-triazol. As EBN contain high amount of protein, a few amino acids and peptides such as threonine, proline, leucine and valine were also detected in the sample. Secondary metabolites such as octylamine, cascarillin, roscovitine, terbutaline, lupinine and N-(octadecanoyl)-1-beta-glucosyl-sphinganine were also found in HBN sample.

**TABLE 1:** Metabolites detected from hydrolyzed birds nest (HBN) using LCMSQTOF.

No	Retention time (min)	Ion	Mass	Molecular formula	Tentative identification
1	4.885	[M + H]^+^	293.1120	C_11_H_19_NO_8_	N-Acetylmuramic acid
2	4.905	[M + H]^+^	309.1069	C_11_H_19_NO_9_	N-Acetylneuraminic acid
3	4.906	[M + H]^+^	291.0964	C_11_H_17_NO_8_	N-Acetyl-2,7-anhydro-alpha-neuraminic acid
4	4.927	[M + H]^+^	203.0812	C_9_H_9_N_5_O	3-Phenyl-5-ureido-1,2,4-triazol
5	5.317	[M + H]^+^	216.111	C_9_H_16_N_2_O_4_	Threonyl-proline
6	5.456	[M + H]^+^	115.0641	C_5_H_9_NO_2_	D-Proline
7	5.897	[M + H]^+^	129.1519	C_8_H_19_N	Octylamine
8	6.182	[M + H]^+^	408.2143	C_22_H_22_N_2_O_2_	Cascarillin
9	6.419	[M + H]^+^	230.1633	C_11_H_22_N_2_O_3_	L-Leucyl-L-Valine
10	7.378	[M + H]^+^	354.2169	C_19_H_26_N_6_O	Roscovitine
11	7.789	[M + H]^+^	225.137	C_12_H_19_NO_3_	Terbutaline
12	8.475	[M + H]^+^	132.0427	C_5_H_8_O_4_	2-Acetolactic acid
13	8.657	[M+2H]^+2^	618.410	C_32_H_54_N_6_O_6_	Unknown 1
14	9.336	[M + H]^+^	385.2578	C_19_H_35_N_3_O_5_	Actinonin
15	11.439	[M+2H]^+2^	580.391	C_40_H_52_O_3_	(3S,4R,3′R)-4-hydroxyalloxanthin
16	14.863	[M + H]^+^	169.1472	C_10_H_19_NO	Lupinine
17	20.028	[M + H]^+^	729.6106	C_42_H_83_NO_8_	N-(octadecanoyl)-1-beta-glucosyl-sphinganine
18	22.913	[M + H]^+^	462.3184	C_28_H_46_O_3_S	Vitamin D3
19	24.681	[M + H]^+^	427.3047	C_22_H_41_N_3_O_5_	Unknown 2
20	25.57	[M + H]^+^	311.2099	C_17_H_29_NO_4_	Unknown 3

### The Effect of HBN on Body Weight

**FIGURE 2:**
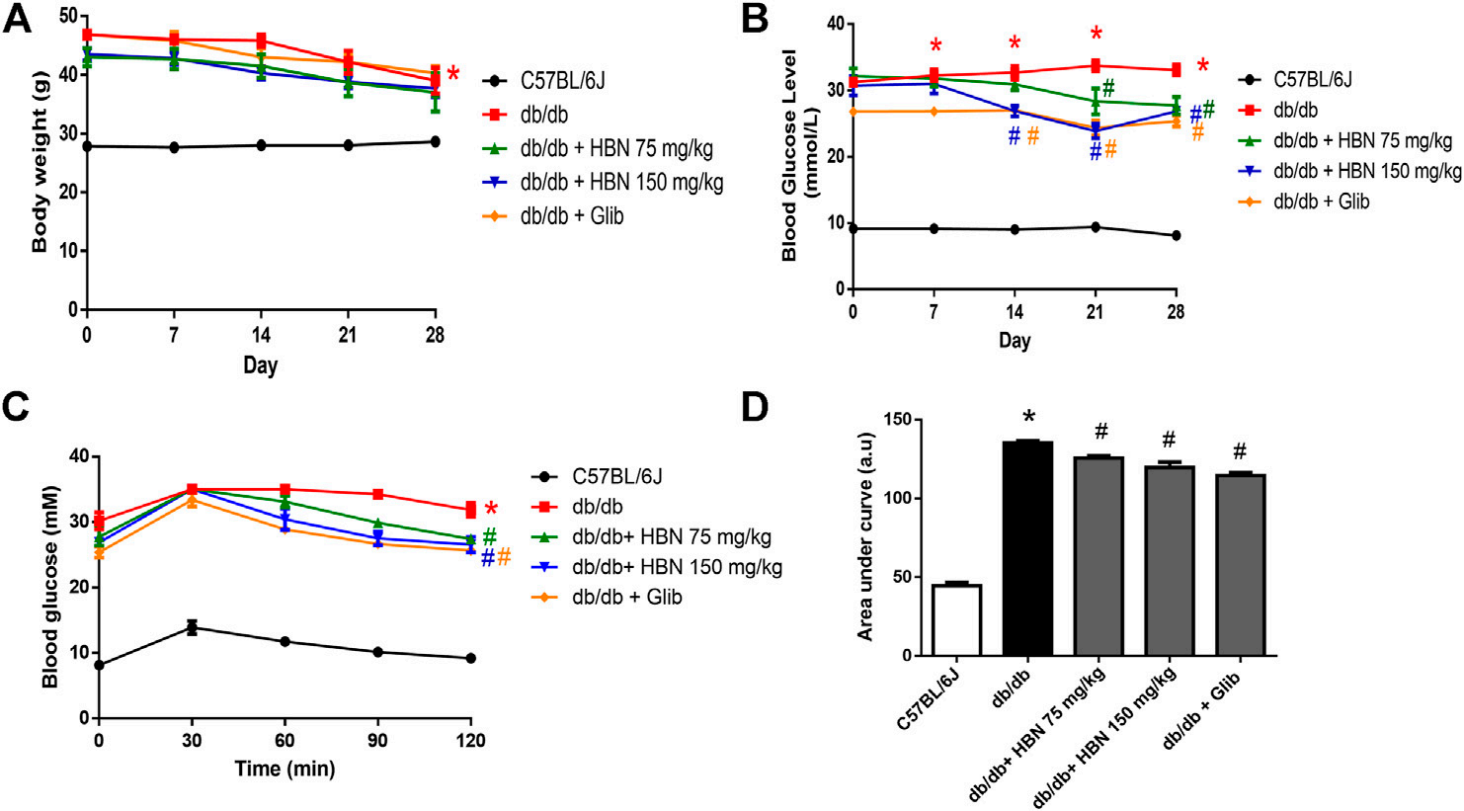
Effect of HBN on body weight, fasting blood glucose and glucose tolerance. **(A)** Weekly body weight and from day 0 to day 28, **(B)** weekly fasting blood glucose **(C)** oral glucose tolerance test (OGTT) and **(D)** area under the curve for OGTT following treatment with HBN (75 and 150 mg/kg) and glibenclamide (1 mg/kg) in *db/db* mice. Results are means ± SEM of six experiments. *p < 0.05 compared to C57BL/6J mice, #*p* < 0.05 compared to *db/db* mice.

The body weight of *db/db* mice were significantly higher than the non-diabetic mice at day 0 (46.8 ± 0.75 vs. 27.8 ± 0.65 g) and remained high until end of treatment at day 28 (39.00 ± 2.19 vs. 28.7 ± 0.61 g). There were no significant differences in the body weights for all *db/db* groups at day 0. The body weight of all *db/db* mice decreased slightly from day 14 onwards. There was no significant difference in body weight between vehicle, HBN or glibencamide treated *db/db* mice at day 28 (Figure 2A).

### The Effect of HBN on Fasting Blood Glucose and Glucose Tolerance

The diabetic groups demonstrated a significantly elevated fasting blood glucose (>20 mM) compared to the non-diabetic control (<10 mM) on day 0 and remained high throughout the 28 days. The *db/db* mice treated with HBN 150 mg/kg demonstrated a significant decrease from day 14th while HBN 75 mg/kg and glibenclamide treated groups started showing a significant decrease from day 21. There was about 20% reduction in blood glucose level at the end of treatment at day 28 for all treated groups compared to *db/db* mice (Figure 2B).

The diabetic groups demonstrated a significantly increased in glucose tolerance compared to the non-diabetic control. Treatment with HBN (75 and 150 mg/kg) and glibenclamide decreased significantly the glucose tolerance by about 7–10% in *db/db* mice (Figures 2C,D). The HBN (150 mg/kg) and glibenclamide treatment showed significant decrease in glucose tolerance starting 60 min after glucose administration and continue to decrease at 90 and 120 min while the treatment with HBN (75 mg/kg) showed significant decrease in glucose tolerance starting 90 min after glucose administration till the end point at 120 min.

### The Effect of HBN on Insulin Expression in Islets of Langerhans and Serum Insulin

**FIGURE 3:**
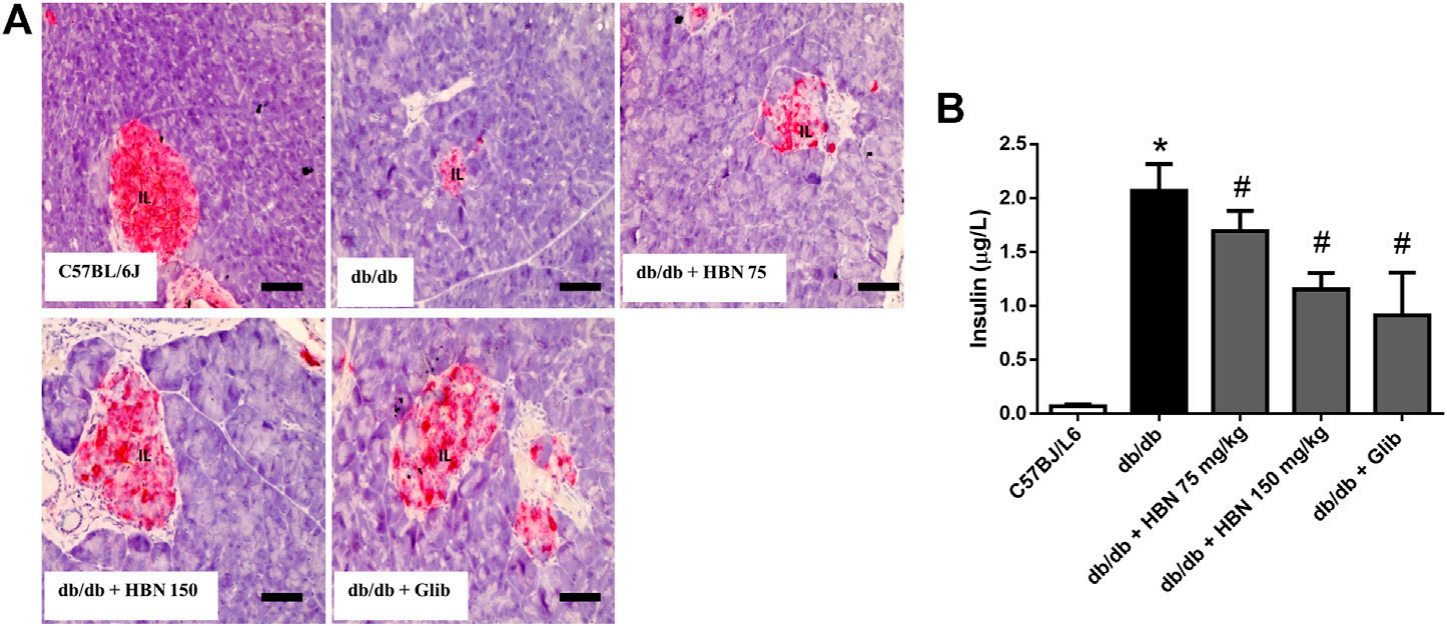
Effects of HBN (75 and 150 mg/kg) and glibenclamide (1 mg/kg) treatment on insulin content. **(A)** Representative immunoperoxidase images showing insulin distribution in the pancreatic islets. The sites of distribution are indicated by dark brown staining. Scale bar = 50 μm. Magnification ×40 **(B)** Serum insulin level (µg/L). Values are expressed as mean ± S.E.M of six experiments. *p < 0.05 compared to C57BL/6J mice, #*p* < 0.05 compared to *db/db* mice.

Insulin expression was significantly reduced in pancreatic islets of *db/db* mice compared to control group. In diabetic rats treated with HBN (75 and 150 mg/kg), increased insulin expression was observed. Higher insulin expression was observed with 150 mg/kg HBN treatment. Treatment with glibenclamide also significantly increased pancreatic insulin content (Figure 3A). Serum insulin were markedly elevated in *db/db* mice indicating hyperinsulinemia. Supplementation of HBN (75 and 150 mg/kg) and glibenclamide (1 mg/kg) in the *db/db* mice significantly decreased the elevated serum insulin (Figure 3B).

### The Effect of HBN Treatment on Pro-inflammatory Cytokines

**FIGURE 4:**
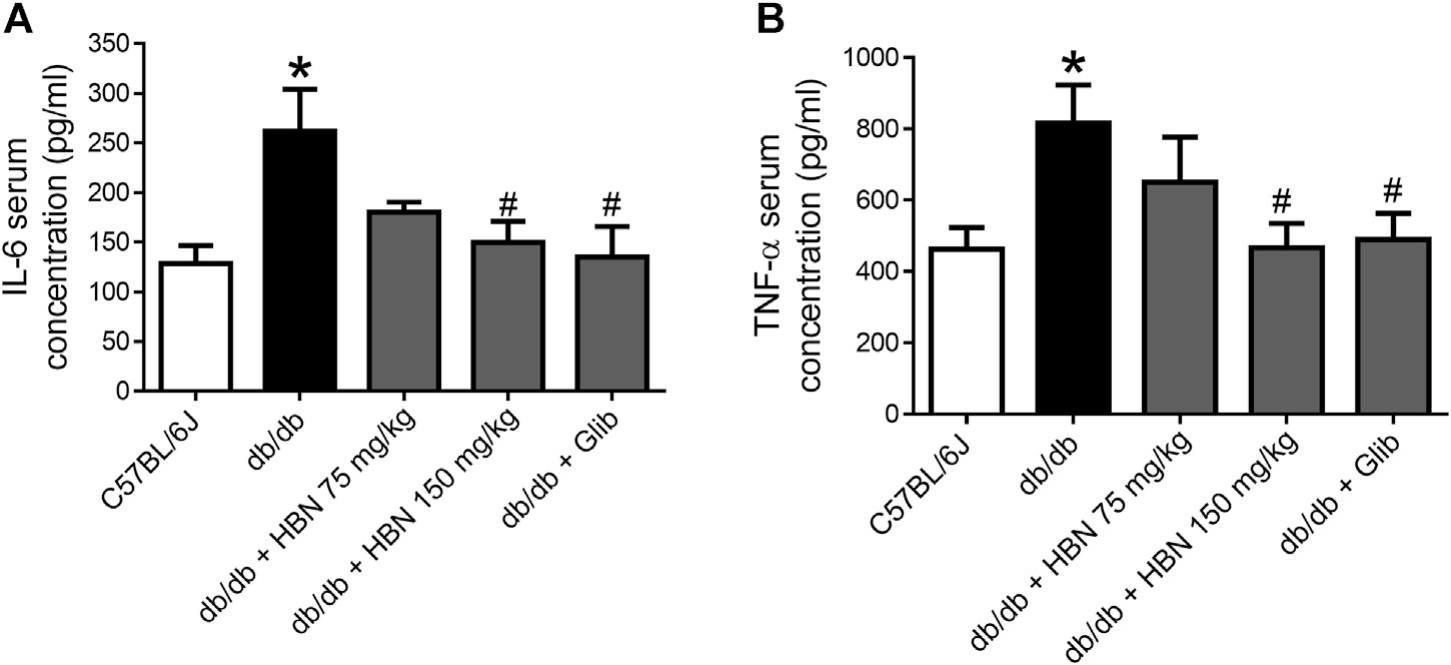
Effects of HBN on serum pro-inflammatory cytokines. The level of pro-inflammatory cytokines of **(A)** IL-6 and **(B)** TNF-α in blood serum in C57BJ/L6 mice and *db/db* mice after treatment with HBN (75 and 150 mg/kg) and glibenclamide (1 mg/kg). Results are mean ± SEM of six experiments. *p < 0.05 compared to C57BL/6J mice, #*p* < 0.05 compared to *db/db* mice.

Pro-inflammatory cytokines, IL-6 and TNF-α were increased by two folds in the serum of *db/db* mice. Treatment with HBN (150 mg/kg) and glibenclamide significantly reduced the production of pro-inflammatory cytokines of IL-6 and TNF-α. Although not significant, treatment with 75 mg/kg HBN also demonstrated a decrease in serum IL-6 and TNF-α levels in *db/db* mice (Figures 4A,B).

### The Effect of HBN on Histopathological Changes of Pancreas and Liver

Islet of Langerhans appeared small and degranulated in the diabetic group compared to control group. The acinar cells were swollen, and small vacuoles were observed in almost all acinar cells. Interlobular ducts were lined with flattened epithelium. However, in diabetic rats treated with HBN extracts or glibenclamide, the islets were bigger and less necrotic (Supplementary Figure S1A). The liver from normal control mice showed normal histological structure, regular distinct hepatocytes with sinusoidal spaces arranged radially around the central vein. The diabetic group showed some fatty changes with necrosis and necrobiosis in the hepatocytes. However, diabetic mice treated with HBN (75 and 150 mg/kg) and glibenclamide showed decreased fatty changes with lower necrosis (Supplementary Figure S1B).

### Effect of HBN on Insulin Signaling, Inflammatory and Oxidative Stress Proteins in Liver and Adipose Tissues

**FIGURE 5:**
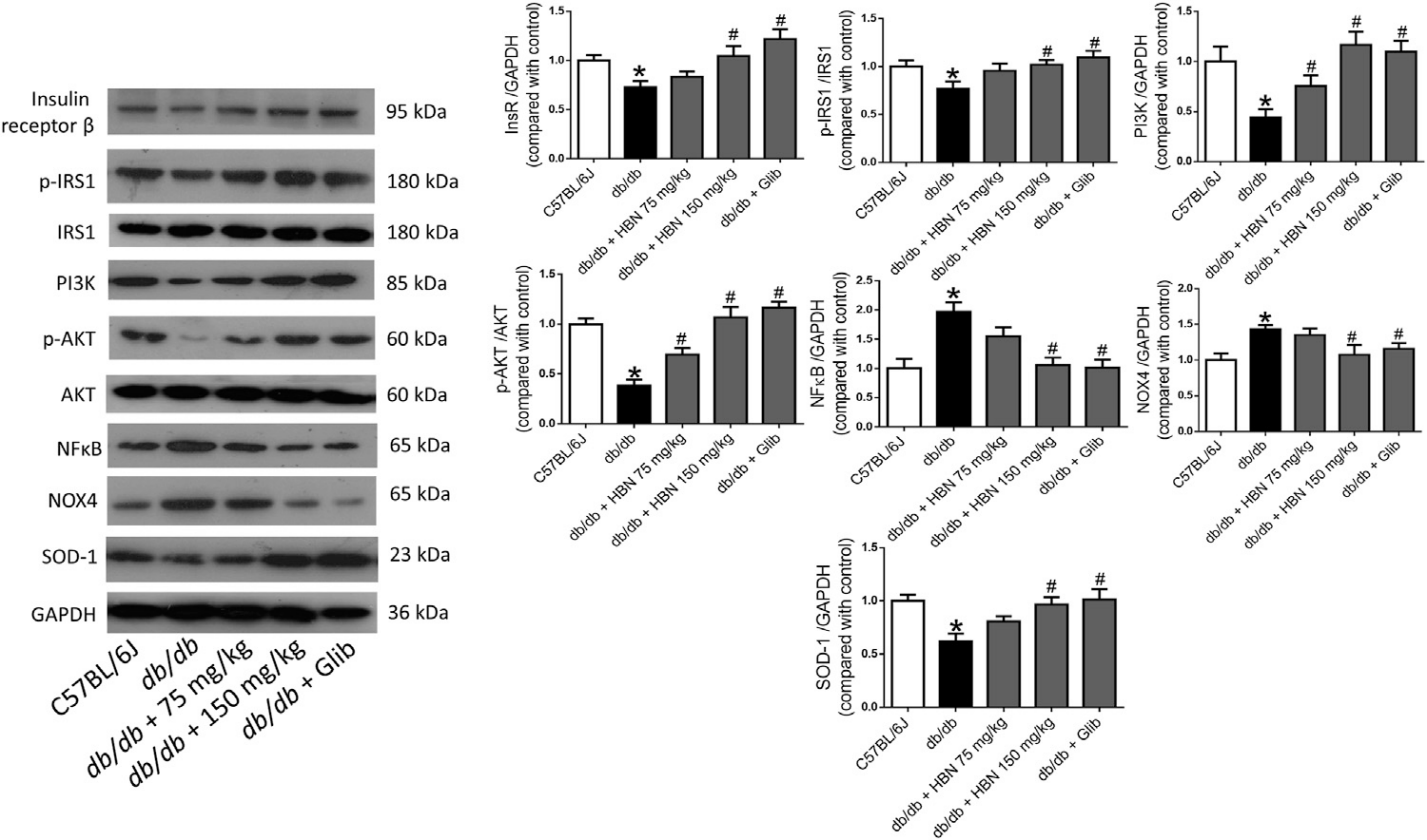
Effects of HBN on hepatic insulin signaling, inflammation and oxidative stress. Western blot and quantitative data showing the protein expression in liver of C57BJ/L6 mice and *db/db* mice after treatment with HBN (75 and 150 mg/kg) and glibenclamide (1 mg/kg). Results are mean ± SEM of six experiments. *p < 0.05 compared to C57BL/6J mice, #*p* < 0.05 compared to *db/db* mice.

**FIGURE 6:**
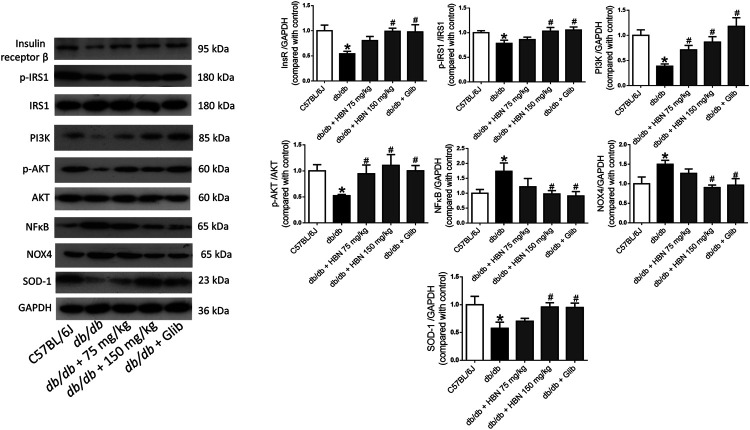
Effects of HBN on adipose insulin signaling, inflammation and oxidative stress. Western blot and quantitative data showing the protein expression in adipose tissue of C57BJ/L6 mice and *db/db* mice after treatment with HBN (75 and 150 mg/kg) and glibenclamide (1 mg/kg). Results are mean ± SEM of six experiments. *p < 0.05 compared to C57BL/6J mice, #*p* < 0.05 compared to *db/db* mice.

In liver (Figure 5) and adipose tissue (Figure 6), the protein expression of insulin receptors (IRβ) and the downstream proteins of insulin signaling, p-IRS1, PI3K and p-Akt was downregulated in the *db/db* mice compared to non-diabetic mice. Treatment of HBN (150 mg/kg) and glibenclamide in the diabetic mice upregulated the IRβ, p-IRS1, PI3K and p-Akt in both liver and adipose. Treatment with HBN (75 mg/kg) in liver and adipose tissue significantly upregulated PI3K and p-Akt. Although this dose demonstrated a slight upregulation of IRβ and p-IRS1, it was not significant.

In contrast, the inflammatory protein NFkB were upregulated by 1-fold in both the liver (Figure 5) and adipose tissue (Figure 6) of the *db/db* group. The treatment with HBN (75 and 150 mg/kg) and glibenclamide reversed the upregulation of NFkB. Similarly, the reactive oxygen species (ROS) marker, NOX4 protein was upregulated in the liver and adipose tissue of the diabetic group and this upregulation was reversed following treatment with HBN (75 and 150 mg/kg) and glibenclamide. In contrast, the antioxidant protein SOD-1 was downregulated in both liver (Figure 5) and adipose tissue (Figure 6) of the diabetic group. Treatment with HBN (75 and 150 mg/kg) and glibenclamide in the diabetic animals upregulated the decreased protein to control level.

### Effect of HBN on GLUT-2 Expression in the Pancreas and Liver

**FIGURE 7:**
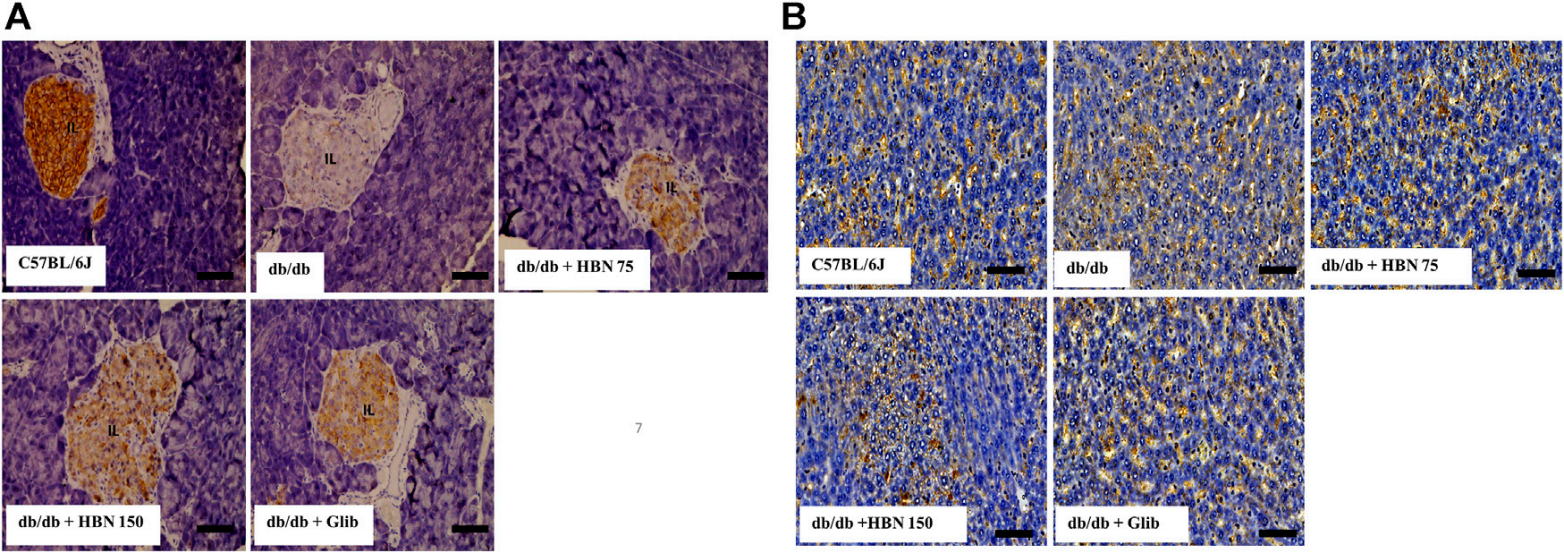
Effects of HBN on glucose transporter 2 (GLUT-2) level. Representative immunoperoxidase images showing GLUT-2 distribution in the pancreas **(A)** and liver **(B)**. The sites of distribution are indicated by dark brown staining. Scale bar = 100 μm. Magnification ×40.

GLUT-2 expression was highest in the pancreatic islets (Figure 7A) and liver (Figure 7B) of non-diabetic group and significantly lower in the pancreatic islets and liver of the diabetic group. Treatment of diabetic mice with HBN (75 and 150 mg/kg) and glibenclamide resulted in higher GLUT-2 expression in the pancreatic islets and liver compared to untreated diabetic group.

### Effect of HBN on GLUT-4 Expression in the Liver and Skeletal Muscle

**FIGURE 8:**
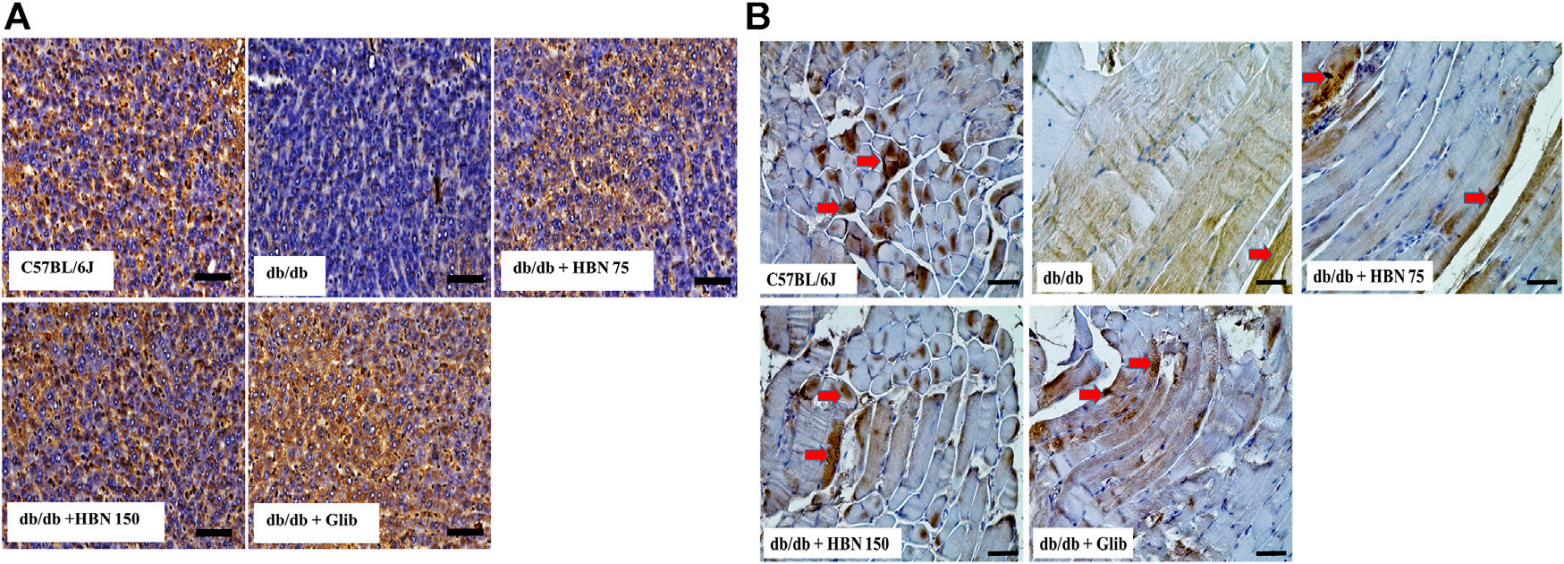
Effects of HBN on glucose transporter 4 (GLUT-4) level. Representative immunoperoxidase images showing GLUT-4 distribution in the liver **(A)** and skeletal muscle **(B)**. The sites of distribution are indicated by dark brown staining. Scale bar = 100 μm. Magnification ×40.

Immunostaining showed lower levels of GLUT-4 protein expression in the liver (Figure 8A) and skeletal muscle (Figure 8B) of *db/db* group compared to the C57BL/6J group. Treatment of diabetic mice with HBN (75 and 150 mg/kg) and glibenclamide showed significantly higher GLUT-4 expression in the liver and skeletal muscle.

## Discussion

The present study demonstrated that oral treatment with HBN exerted anti-diabetic effect and improved glucose tolerance in type 2 diabetic mice by protecting the pancreatic β-cells and improving insulin signaling in the liver and adipose tissue. The anti-diabetic effects of HBN is partially attributed to inhibition of chronic inflammation and oxidative stress.

The major compositions of edible bird nest (EBN) reported are carbohydrate (25.62–31.40%) and protein (60–66%) (Zainab et al., 2013). Ma and Liu (2012) reported that the carbohydrates in EBN consist mostly of sialic acid (9.0%), galactosamine (7.2%), glucosamine (5.3%), galactose (16.9%) and fucose (0.7%). Studies have demonstrated high amount of sialic acid contributed to brain development and learning and memory enhancing ability (Wang and Brand-Miller, 2003; Careena et al., 2018). Similarly, our earlier work demonstrated that improvement by HBN in endothelial dysfunction due to high glucose was comparable to the effects of sialic acid *in vitro* (Murugan et al., 2020). Three types of sialic silic acid were determined in HBN. They are N-Acetylmuramic acid, N-Acetylneuraminic acid and N-Acetyl-2,7-anhydro-alpha-neuraminic acid. Normally the sialic acid exist in conjugated form such as oligosaccharides and glycoprotien. However, after hydrolysis of EBN, the neuraminic acid or acetylmuramic acid will be released (Chan et al., 2013).

The *db/db* mice model is one of the most frequently used models to emulate type 2 diabetes in humans. *db/db* mouse lacks leptin receptor which makes them susceptible to obesity, insulin resistance, and type 2 diabetic mellitus (Burke et al., 2017). Our result showed that treatment with HBN and antidiabetic agent, glibencamide did not have significant effect on the body weight of diabetic mice. However, treatment with HBN in diabetic mice significantly decreased the fasting blood glucose starting from day 7 of treatment for the higher dose (150 mg/kg) and day 14 for the lower dose (75 mg/kg). Both doses also improved glucose tolerance further supporting the anti-diabetic effect of HBN. According to Zulkifli et al., 2019, hydrolysis of EBN lead in improving the functional properties of EBN and result in the increasing of antioxidant and anti-hyperglycemic activities which is agreeable with the present study which used hydrolyzed edible bird’s nest.

Insulin is produced in pancreatic β-cells and is packaged into membrane-bound secretory granules which is released upon stimulation of β-cells by glucose or other secretagogues (Do and Thorn, 2015). We have demonstrated that supplementation with HBN in diabetic mice preserved pancreatic functions by restoring the histopathological changes in the pancreas as observed by the near normal pancreatic islets and β-cell numbers. Furthermore, HBN treatment reduced hyperinsulineamia in the diabetic group in an attempt to normalize the plasma insulin level.

Persistent hyperglycaemia in diabetes mellitus increases the production of reactive oxygen species (ROS) as well as suppressing antioxidant defense mechanisms which ultimately contribute to oxidative stress (Jha et al., 2018). Nicotinamide adenine dinucleotide phosphate (NADPH) oxidase (NOX) is a multicomponent enzyme complex that produces reactive oxygen species (ROS) (Yong et al., 2013). Among the NOX isoforms, NOX4 is associated with diabetes where it dysregulates stress signaling, fibrosis, and insulin sensitivity in hepatocytes and Kupffer cells of liver (Bettaieb et al., 2015) and adipocytes (Den Hartigh et al., 2017). In addition, a decrease in potent antioxidants such as superoxide dismutase (SOD), catalase (CAT) and the glutathione (GSH) enzyme family during hyperglycaemic condition will cause an increase in ROS, which eventually contributes to oxidation-induced liver and adipose tissue damages (Mohamed et al., 2016; Maslov et al., 2019). The present results showed that the level of NOX4 was elevated while SOD-1 was reduced in liver and adipose tissue of *db/db* mice, which were all reversed with the supplementation of 150 mg/kg HBN.

In addition, increased level of inflammation is noted in the *db/db* mice as evidenced by increased level of NFKB protein expression in the liver and adipose tissue and increased serum cytokines, IL-6 and TNF-α. Translocation of NFKB into the nucleus increases the expression of inflammatory cytokines such as TNF-α and IL-1β (Liu et al., 2017). The marked reduction of serum TNF-α and IL-6 following 150 mg/kg HBN supplementation reduces inflammation and help to protect the liver, pancreas and adipose tissue from the hyperglycaemic insults. Similar anti-inflammatory and antioxidant effects of edible bird's nest have been demonstrated in high fat diet induced obese rats (Yida et al., 2015) and following exposure to elevated glucose levels *in vitro* to human SH-SY5Y cells (Yew et al., 2014; Hou et al., 2015) and in isolated *db/db* aorta (Murugan et al., 2020).

Chronic inflammation play an important role in the pathogenesis of insulin resistance, the hallmark of type 2 diabetes mellitus (Rehman and Akash, 2016). Activation of TNF-α triggers insulin resistance by increasing uptake of glucose in visceral and subcutaneous adipocytes (Fernandez-Veledo et al., 2009) and through phosphorylation of serine 307 in IRS-1 (Aguirre et al., 2000). It has been reported that IL-1β amplified systemic inflammation and impaired insulin signaling leading to insulin resistant (Boni-Schnetzler and Donath, 2013) and organ dysfunction (Grant and Dixit, 2013) in peripheral tissues and macrophages during type 2 diabetes mellitus. Both TNF-α and IL-1β downregulation insulin receptor β in diabetic insulin-resistant hepatocytes and adipocytes (Jager et al., 2007; Alipourfard et al., 2019).

Phosphorylated IRS proteins activates PI3K/Akt signaling pathway demonstrating its role in the metabolic action of insulin (Huang et al., 2018). In adipose tissue, insulin-Akt signaling promotes utilization of glucose, gluconeogenesis and lipid biosynthesis. In liver, it has been reported that PI3K/Akt signaling pathway attenuates production of hepatic glucose and glycogenolysis, increases synthesis of glycogen and fatty acids for storage and subsequent utilization by other tissues (Huang et al., 2018). Similarly, the current study showed that insulin signaling proteins such as insulin receptor β, phosphorylation of IRS, PI3K and phosphorylation of Akt in liver and adipose tissues were significantly downregulated in *db/db* mice, suggesting that the insulin signaling was impaired in type 2 diabetic animal. Treatment with 150 mg/kg HBN in *db/db* mice improved these metabolic changes associated with dysregulation of insulin signaling.

It has been reported that level of Akt and mobility of glucose transporter in the vesicles/membrane is closely related (Kuai et al., 2016). Akt catalyses the AS160 substrate protein phosphorylation which triggers GLUT glucose transporters translocation from cytoplasmic vesicles onto the cell membrane surface which facilitate the insulin-dependent transport of glucose into the cell (Kuai et al., 2016). GLUT-2 and GLUT-4, the isoforms of glucose transporters have essential roles in mediating disposal of body glucose. GLUT-2, unique among the facilitative hexose transporters due to its low affinity and high capacity for glucose, is expressed primarily in cells involved in glucose sensing, such as hepatocytes and pancreatic β cells. When plasma glucose concentrations are elevated, which are typical of the postprandial state, GLUT-2 responds with rapid, continual net uptake of glucose for insulin secretion (Kuai et al., 2016).

Meanwhile, the majority of peripheral glucose uptake into adipose tissue and skeletal muscle is achieved through similar signal transduction pathways and is mediated by the insulin responsive GLUT-4 facilitative glucose transporter (Stringer et al., 2015). GLUT-4 disruption in adipose or muscle tissue will cause insulin resistance and thus promote higher risk for diabetes (Abel et al., 2001). In the present study, GLUT-2 and GLUT-4 were decreased in the diabetic mice, and treatment with HBN increased the expression of both glucose transporters. The increase in GLUT-2 level with HBN treatment was more prominent in the pancreas than in the liver. On the other hand, the expression of GLUT-4 level was similarly increased in the liver and skeletal muscle of the HBN-treated diabetic groups. This finding suggest that increased in GLUT-2 receptors subsequently promote uptake of glucose into the β-cells to stimulate insulin secretion by the pancreas while GLUT-4 promotes uptake of glucose to tissues.

In conclusion, the present findings suggest that oral supplementation of HBN exhibited anti-diabetic effect in type 2 diabetic *db/db* mice as evidenced by the improvement of β-cell function and insulin sensitivity *via* the insulin-PI3K/Akt signaling pathway. This is potentially achieved through reduction in oxidative stress and chronic inflammation. HBN may be useful as a nutraceutical supplement in improving glucose control in type 2 diabetes mellitus.

## Data Availability

The datasets generated for this study are available on request to the corresponding author.
