# Supplementary figures and images for: Effect of Hydrolyzed Bird’s Nest on β-Cell Function and Insulin Signaling in Type 2 Diabetic Mice
Source: Front Pharmacol. 2021 Apr 13;12:632169. doi: 10.3389/fphar.2021.632169 (PMC8112233; doi:10.3389/fphar.2021.632169)

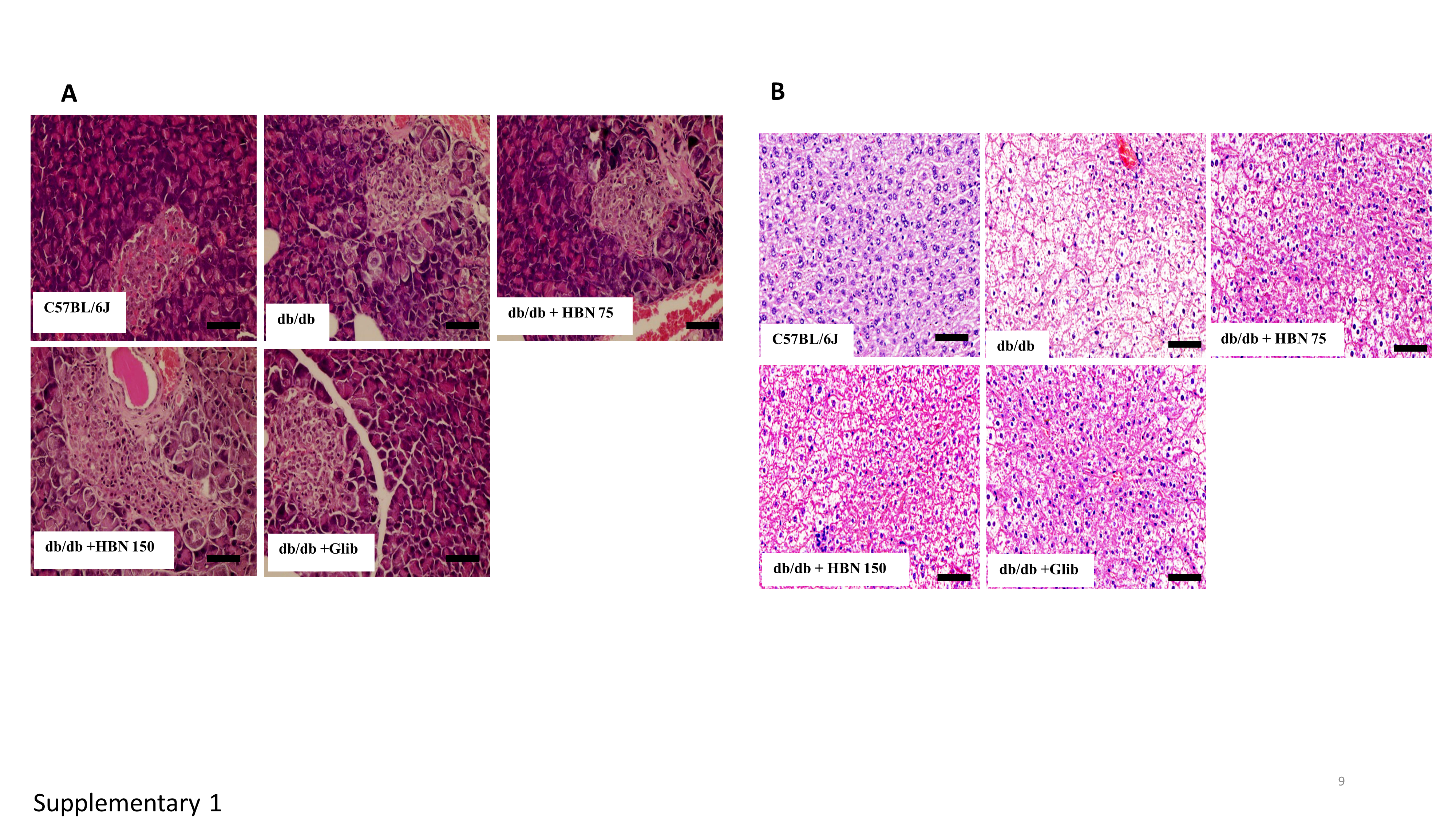

Supplement: Supplementary file 1 [file image1.tif]
