# Supplementary material for: Effect of Hydrolyzed Bird’s Nest on β-Cell Function and Insulin Signaling in Type 2 Diabetic Mice
Source: Front Pharmacol. 2021 Apr 13;12:632169. doi: 10.3389/fphar.2021.632169 (PMC8112233; doi:10.3389/fphar.2021.632169)
Supplement: Supplementary file 2 [file image2.tif]

Frontiers | Effect of Hydrolyzed Bird's Nest on β-cell function and insulin signaling in type 2 diabetic mice | Pharmacology


- About
- Journals
- Research Topics
- Articles
- More

Submit

My Frontiers

Office

- TSOF
  - TSOF
  - Article Production

Typesetter 3

frontiersproduction@tnq.co.in

- Profile
- Settings & Privacy
- Help Center
- Logout

Submit

**Impact Factor 4.225** | **CiteScore 5.0**More on impact ›

|  |  |
| --- | --- |
| Frontiers in Pharmacology | Ethnopharmacology |

Toggle navigation


Section


- (current)Section
- About
- Articles
- Research topics
- For authors 
  - Why submit?
  - Fees
  - Article types
  - Author guidelines
  - Review guidelines
  - Submission checklist
  - Contact editorial office
  - Submit your manuscript
- Editorial board

- *Article alerts*

##### This article is part of the Research Topic

Edible Birds Nest - Chemical Composition and Potential Health Efficacy and Risks
View all
5
Articles

Articles


**Suggest a Research Topic >**

- 79
  total views

 View Article Impact

**Suggest a Research Topic >**

##### SHARE ON

- Facebook

  0
- Twitter

  0
- LinkedIn

  0
- AddThis

  New


## Original Research ARTICLE

Front. Pharmacol.
| doi: 10.3389/fphar.2021.632169

# Effect of Hydrolyzed Bird's Nest on β-cell function and insulin signaling in type 2 diabetic mice Provisionally accepted The final, formatted version of the article will be published soon. **Notify me**

Woon Ker1, Zuhaida M. Zain2, 
 Dharmani Devi Murugan2\*, Giribabu Nelli2, 
Nor Hisam Zamakshshari2, 
 Yang Mooi Lim3 and  Mohd R. Mustafa2

- 1Universiti Teknologi MARA Puncak Alam, Malaysia
- 2University of Malaya, Malaysia
- 3Tunku Abdul Rahman University College, Malaysia

Type 2 diabetes mellitus is characterized by both resistance to the action of insulin and defects in insulin secretion. Bird's nest, which is derived from the saliva of swiftlets are well known to possess multiple health benefits dating back to Imperial China. However, it’s effect on diabetes mellitus and influence on the actions of insulin action remains to be investigated. In the present study, the effect of standardised aqueous extract of hydrolyzed edible bird nest (HBN) on metabolic characteristics and insulin signaling pathway in pancreas, liver and skeletal muscle of db/db, a type 2 diabetic mice model was investigated. Male db/db diabetic and its euglycemic control, C57BL/6J mice were administered HBN (75 mg/kg and 150 mg/kg) or glibenclamide (1 mg/kg) orally for 28 days. Metabolic parameters were evaluated by measuring fasting blood glucose, serum insulin and oral glucose tolerance test (OGTT). Insulin signaling and activation of inflammatory pathways in liver, adipose, pancreas and muscle tissue were evaluated by Western blotting and immunohistochemistry. Pro-inflammatory cytokines were measured in the serum at the end of the treatment. The results showed that db/db mice treated with HBN significantly reversed the elevated fasting blood glucose, serum insulin, serum pro-inflammatory cytokines levels and the impaired OGTT without affecting the body weight of the mice in all groups. Furthermore, HBN treatment significantly ameliorated pathological changes and increased the protein expression of insulin, and glucose transporters in the pancreatic islets (GLUT-2), liver and skeletal muscle (GLUT-4). Likewise, the Western blots analysis denotes improved insulin signaling and antioxidant enzyme, decreased reactive oxygen species producing enzymes and inflammatory molecules in the liver and adipose tissues of HBN treated diabetic mice. These results suggest that HBN improves β-cell function and insulin signaling by attenuation of oxidative stress mediated chronic inflammation in the type 2 diabetic mice.

Keywords: 
Hydrolyzed Bird Nest, type 2 diabetes mellitus, insulin signalling, Oxidative Stress, Inflammation

Received: 22 Nov 2020;
Accepted: 22 Feb 2021.

Copyright: © 2021 Ker, Zain, Murugan, Nelli, Zamakshshari, Lim and Mustafa. This is an open-access article distributed under the terms of the Creative Commons Attribution License (CC BY). The use, distribution or reproduction in other forums is permitted, provided the original author(s) and the copyright owner(s) are credited and that the original publication in this journal is cited, in accordance with accepted academic practice. No use, distribution or reproduction is permitted which does not comply with these terms.

\* Correspondence: 
Dr. Dharmani Devi Murugan, University of Malaya, Kuala Lumpur, 50603, Malaysia, dharmani79@um.edu.my

Write a comment...

Add

##### COMMENTARY

##### ORIGINAL ARTICLE

##### People also looked at

## Chemoresponse of de novo acute myeloid leukemia to "7+3" induction can be predicted by c-Myc-facilitated cytogenetics

Tzu-Hung Hsiao, Ren Ching Wang, Tsai-Jung Lu, Chien-Hung Shih, Yu-Chen Su, Jia-Rong Tsai, Pei-Pei Jhan, Cai-Sian Lia, Han-Ni Chuang and Chieh-Lin Jerry Teng

## Edible Bird’s Nest Protects Against Hyperglycemia-Induced Oxidative Stress and Endothelial Dysfunction

Dharmani Devi Murugan, Zuhaida Md Zain, Ker Woon Choy, Nor Hisam Zamakshshari, Mel June Choong, Yang Mooi Lim and Mohd Rais Mustafa

## Anti-Hypertensive Herbs and Their Mechanisms of Action: Part II

M. Akhtar Anwar, Sara S. Al Disi and Ali H. Eid

## Immunomodulatory effects and mechanisms of Curcuma species and their bioactive compounds: a review

Yuandani -, Ibrahim Jantan, Ade Sri Rohani and Imam Bagus Sumantri

## The Potential Therapeutic Effect of RNA Interference and Natural Products on COVID-19: A Review of the Coronaviruses Infection

Mohammad Reza Kalhori, Fatemeh Saadatpour, Ehsan Arefian, Masoud Soleimani, Mohammad Hosien Farzaei, Ina Yosifova Aneva and Javier Echeverría

**Suggest a Research Topic >**

×

#### Supplementary Material

  

There is no supplementary material currently available for this article

Loading supplemental data...

  

|  | File Name |  |
| --- | --- | --- |
|  | Image 1.TIF |  |
|  | Image 2.TIF |  |

  

Close

- About Frontiers
- Institutional Membership
- Books
- News
- Frontiers' social media
- Contact
- Careers
- Submit
- Newsletter
- Help Center
- Terms & Conditions
- Privacy Policy

© 2007 - 2021 Frontiers Media S.A. All Rights Reserved

### Privacy Preference Center

Our website uses cookies that are necessary for its operation. Additional cookies are only used with your consent. These cookies are used to store and access information such as the characteristics of your device as well as certain personal data (IP address, navigation usage, geolocation data) and we process them to analyse the traffic on our website in order to provide you a better user experience, evaluate the efficiency of our communications and to personalise content to your interests. Some cookies are placed by third-party companies with which we work to deliver relevant ads on social media and the internet. Click on the different categories' headings to change your cookie preferences. Click on "More Information" if you wish to learn more about how data is collected and shared.
More information

### Manage Consent Preferences

#### Strictly Necessary Cookies

Always Active

These cookies are necessary for the website to function and cannot be switched off in our systems. They are usually only set in response to actions made by you which amount to a request for services, such as setting your privacy preferences, logging in or filling in forms. You can set your browser to block or alert you about these cookies, but some parts of the site will not then work. These cookies do not store any personally identifiable information.

#### Analytics Cookies

Analytics Cookies

These cookies allow us to count visits and traffic sources so we can measure and improve the performance of our site. They help us analyse which pages are the most and least popular and see how visitors move around the site.    All information these cookies collect is aggregated and therefore anonymous.

#### Functional Cookies

Functional Cookies

These cookies enable the website to provide enhanced functionality and personalisation. They may be set by us or by third party providers whose services we have added to our pages. If you do not allow these cookies then some or all of these services may not function properly.

#### Advertising Cookies

Advertising Cookies

These cookies may be set through our site by our advertising partners. They may be used by those companies to build a profile of your interests and show you relevant adverts on other sites.    They do not store directly personal information, but are based on uniquely identifying your browser and internet device. If you do not allow these cookies, you will experience less targeted advertising.

### Back Button Performance Cookies

Vendor Search  Search Icon

Filter Icon

Clear

checkbox label label

Apply Cancel

Consent Leg.Interest

checkbox label label

checkbox label label

checkbox label label

Confirm My Choices
